# Long Prayers

**Published:** 1855-05

**Authors:** 


### LONG PRAYERS

Are impolitic—they engender an irritable frame of mind, and make the body restless. Short, earnest, fervent prayers wake up the attention and soften and soothe the unquiet spirit. How it is with others I do not pretend to say; but this I know for myself, that when I am compelled to listen to a very long prayer, instead of joining in with the petitioner, I am all the time praying that he would quit. I know it is very wrong to do so; but it steals over me before I am aware of it, and leads me into another wrong doing, that of feeling more thankful that the prayer is over, than for blessings from above. Long prayers are for the closet, for the secret chamber, where none can witness but the All-Seeing Eye.

